# Case report: necrotizing enterocolitis with a transverse colonic perforation in a 2-day old term neonate and literature review

**DOI:** 10.1186/s40748-020-00124-0

**Published:** 2021-01-07

**Authors:** Jo-Anna Hudson, Simon Byrns, Elizabeth Nizalik, Emanuela Ferretti

**Affiliations:** 1 Department of Pediatrics, Division of Neonatology, University of Ottawa, Children’s Hospital of Eastern Ontario, Ottawa, Ontario Canada; 2 Department of Pediatric General Surgery, University of Ottawa, Children’s Hospital of Eastern Ontario, Ottawa, Ontario Canada; 3 Department of Pathology, University of Ottawa, Children’s Hospital of Eastern Ontario, Ottawa, Ontario Canada

**Keywords:** Necrotizing enterocolitis, Term neonate, Colonic perforation, Hematochezia, Case report

## Abstract

**Background:**

Necrotizing enterocolitis (NEC), while classically discussed in preterm and low birth weight neonates, also occurs in the term infant and accounts for 10% of all NEC cases. Despite there being fewer reported cases of NEC in term infants, these presentations demonstrate differences in the onset, severity and risk factors from the classic presentation observed in premature infants. We present a novel case of term NEC that contravenes the reported literature making departures from clinical presentation, risk factors and location of perforation in an otherwise healthy term two-day old infant born after an uncomplicated pregnancy who presented with hematochezia.

**Case presentation:**

A healthy term baby born after an uneventful pregnancy presented with bloody stool at 2 days of life who was otherwise well. Investigations revealed pneumoperitoneum from a large proximal transverse colonic perforation secondary to NEC. No typical risk factors for NEC were found.

**Conclusion:**

Given the life-threatening potential of an unrecognized perforation we recommend the inclusion of NEC on the differential for neonatal hematochezia.

## Introduction

This case highlights the presentation of a term two-day old neonate with bloody stools which has a broad differential ranging from benign to life threatening. Here the underlying aetiology was necrotizing enterocolitis with a large transverse colonic perforation. This unique case comprises a series of rare elements from age at onset of symptoms, to the absence of risk factors, to the underlying pathology in a term infant with the rare complication of intestinal perforation in a location not in keeping with known case reports. This case underscores the importance of maintaining a broad differential as the benign history and physical examination underrepresented the life-threatening nature of the underlying pathology of a large intestinal perforation.

## Case description

A two-day old female born at 39 week’s gestation was referred from a community hospital to a tertiary level neonatal intensive care unit (NICU) with the chief medical concern of bloody stools. Antenatal history was unremarkable. The infant was born after a relatively uncomplicated pregnancy to a 28-year-old G1P0 mother. Maternal serologies for rubella, herpes, HIV and syphilis were protective and group B Streptococcus testing was negative. Maternal cytomegalovirus status was not known. The only maternal medication was prenatal vitamins. There was no history of infections or diabetes. Gestational hypertension developed in the last month of pregnancy did not require intervention. There was no maternal or paternal family history of any gastrointestinal issues, no allergies and no family history of cow’s milk protein allergy (CMPA). Additionally, there was no history of familial bleeding disorders and parents were non-consanguineous. There was no major blood loss at the time of delivery, no bloody discharge from the mother’s nipples and there was no blood loss associated with a neonatal ankyloglossia release performed on the first day of life. The infant was born via spontaneous vaginal delivery with no instrumentation after an induction of labour for pre-eclampsia. Membranes were ruptured for twelve hours and active labour was under two hours. APGARS were 8 and 9 at one and five minutes respectively. The umbilical cord gas had a pH of 7.17 and a base excess of − 7.7 mmol/L. She received standard newborn care including a subcutaneous injection of vitamin K and did not receive any additional medications.

**Fig. 1:**
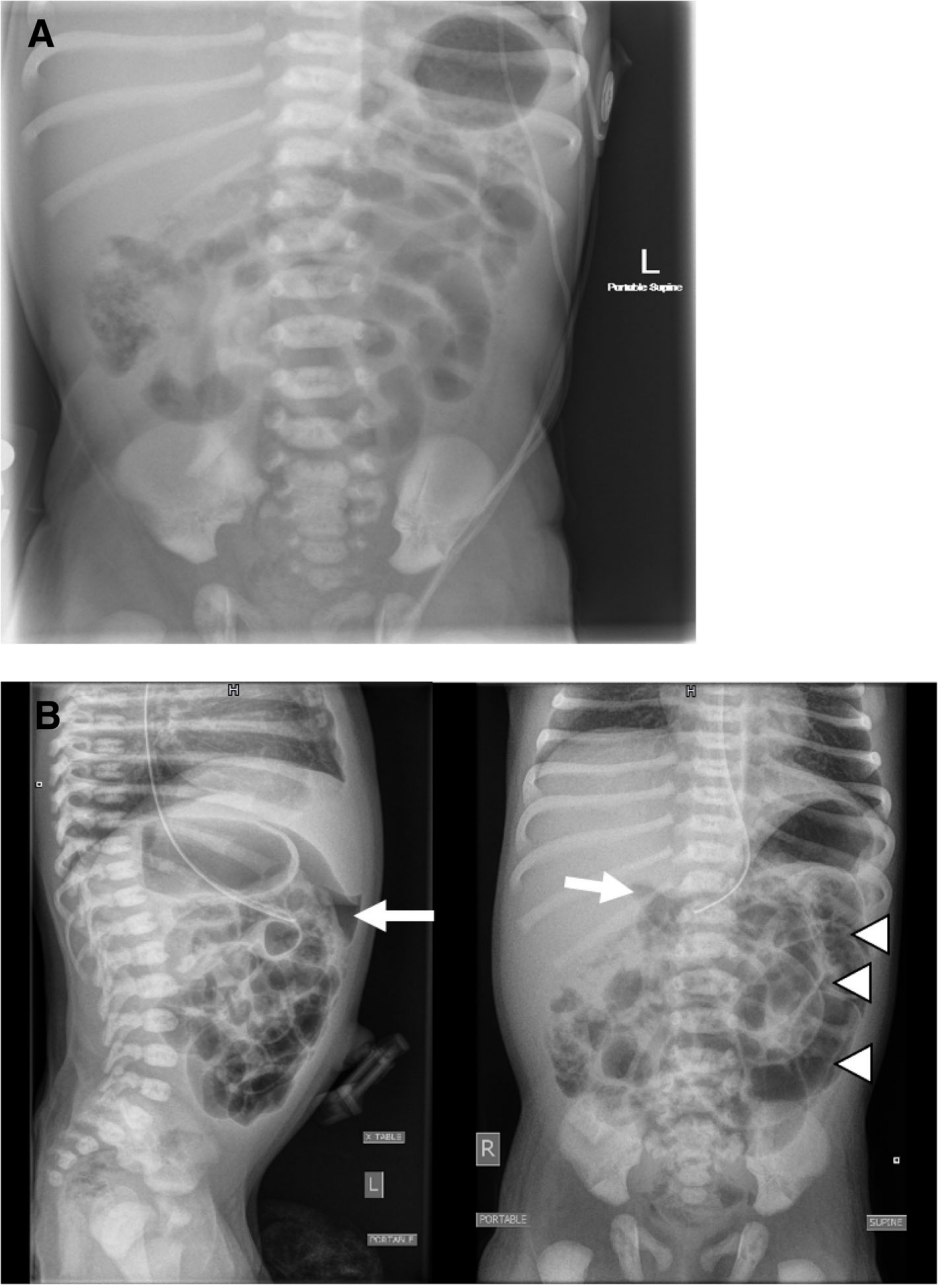
Abdominal X-ray series, performed on the second day of life. **a**. Antero-posterior view taken at ~ 47 h of life. Demonstrating nonspecific gas pattern, no bowel dilation, no pneumatosis, with gas and stool to the level of the rectum. **b**. Antero-posterior and lateral views taken at ~ 55 h of life. Pneumoperitoneum noted inferior to the liver margin and as a triangle lucency over the left side of the abdomen (noted by the arrow) and air outlining both sides of the bowel walls (arrowheads). Mild gaseous distension of several bowel loops more prominent in the left flank and central abdomen. No pneumatosis noted

During the first twenty-four hours of life the infant was breastfeeding with latch issues and required an ankyloglossia release. She subsequently continued to breastfeed and was additionally given formula three times. The family recalled that the newborn had approximately ten dark green to black, loose, foul smelling bowel movements and gradually became less interested in feeding. This was brought to the attention of the physician later the following day. On day of life two initial vitals check at mother’s bedside showed a heart rate of 180 beats per minute and a temperature of 38.3 °C. Upon re-assessment in the nursey the heart rate was 130 beats per minute with a temperature of 37.3 °C and the remainder of her vitals within normal limits. On physical exam baby was reported to be less active but responsive and appropriate with handling, otherwise exam was consistent with a healthy newborn exam; bowel sounds were present, abdomen was soft and there were no hemangiomas present on the infant’s skin. There was a questionable anal fissure at the 12 o’clock position. Initial complete blood counts showed a white cell count of 8 × 10^9^/L, hemoglobin of 19.2 g/dL, platelets of 258 × 10^9^/L and there were no eosinophils present. From a capillary sample, gas and electrolytes were within normal limits; the C-reactive protein was 21.5 mg/L and plasma lactate was 4.2 mmol/L. Over the next ten hours the baby had approximately four moderately bloody stools with blood mixed throughout the stool. Additionally, a coagulation profile was performed and was within normal limits. Blood cultures were drawn (which were subsequently negative) and antibiotics were started. A single view abdominal X-ray was performed (Fig. 1a) that showed a nonspecific bowel gas pattern, without pneumatosis, nor dilated bowel loops. The baby was subsequently transferred uneventfully to our tertiary NICU.

**Fig. 2:**
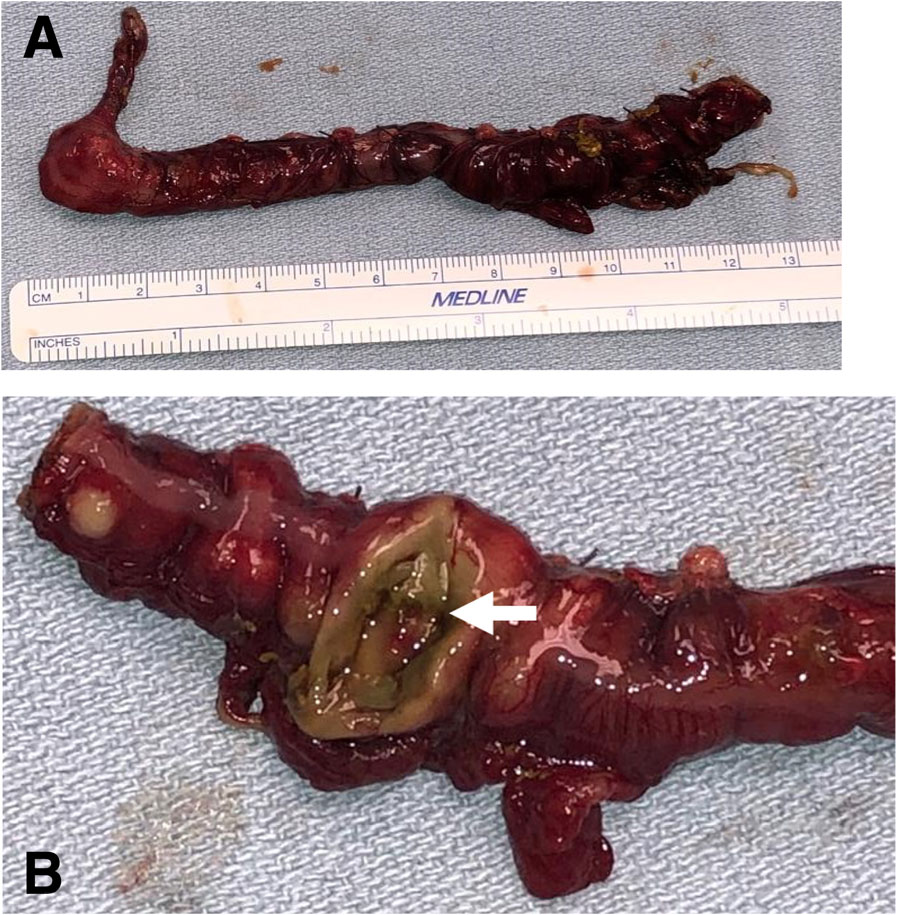
Surgical and macroscopic pathological intestinal tissue sections. **a**. Full pathological specimen – extended right hemicolectomy with appendix on left and perforated transverse colon on right. **b**. Necrotic circular perforation of the transverse colon (arrow) with greenish colour and yellowish colouring at the perforation edges

Upon arrival at the tertiary level NICU, at approximately 55 h of life, while hemodynamically stable on room air, the baby started to produce copious bilious emesis without exhibiting signs of discomfort. Abdominal x-ray (Fig. 1b) showed pneumoperitoneum. Repeat blood work showed a white cell count of 8.8 × 10^9^/L, hemoglobin of 16.4 g/dL, platelets of 200 × ^9^/L and a hematocrit of 46.2%, INR of 1.44, lactate 1.9 mmol/L and a CRP of 83 mg/L. The infant was urgently transferred to the operating room where laparotomy revealed a full thickness perforation of the anterior antimesenteric transverse colon that was two centimeters in diameter, in the mid transverse colon (Fig. 2). There were patches of green-grey discoloration and suspected full thickness necrosis extending to the cecum and appendix suggesting the pathological epicentre was in the mid transverse colon with additional satellite lesions radiating outward. There was no evidence of intrabdominal calcifications to suggest a remote intrauterine perforation. There were no ischemic or necrotic lesions in the distal transverse colon, descending colon, or the sigmoid colon and rectum. Based on these findings, as the etiology was thought to affect the entire ileocolic vascular distribution, an extended right hemicolectomy was performed, with formation of an end ileostomy and distal transverse colon mucous fistula. During resection, no evidence of vascular malformation or anomalies was noted.

**Fig. 3:**
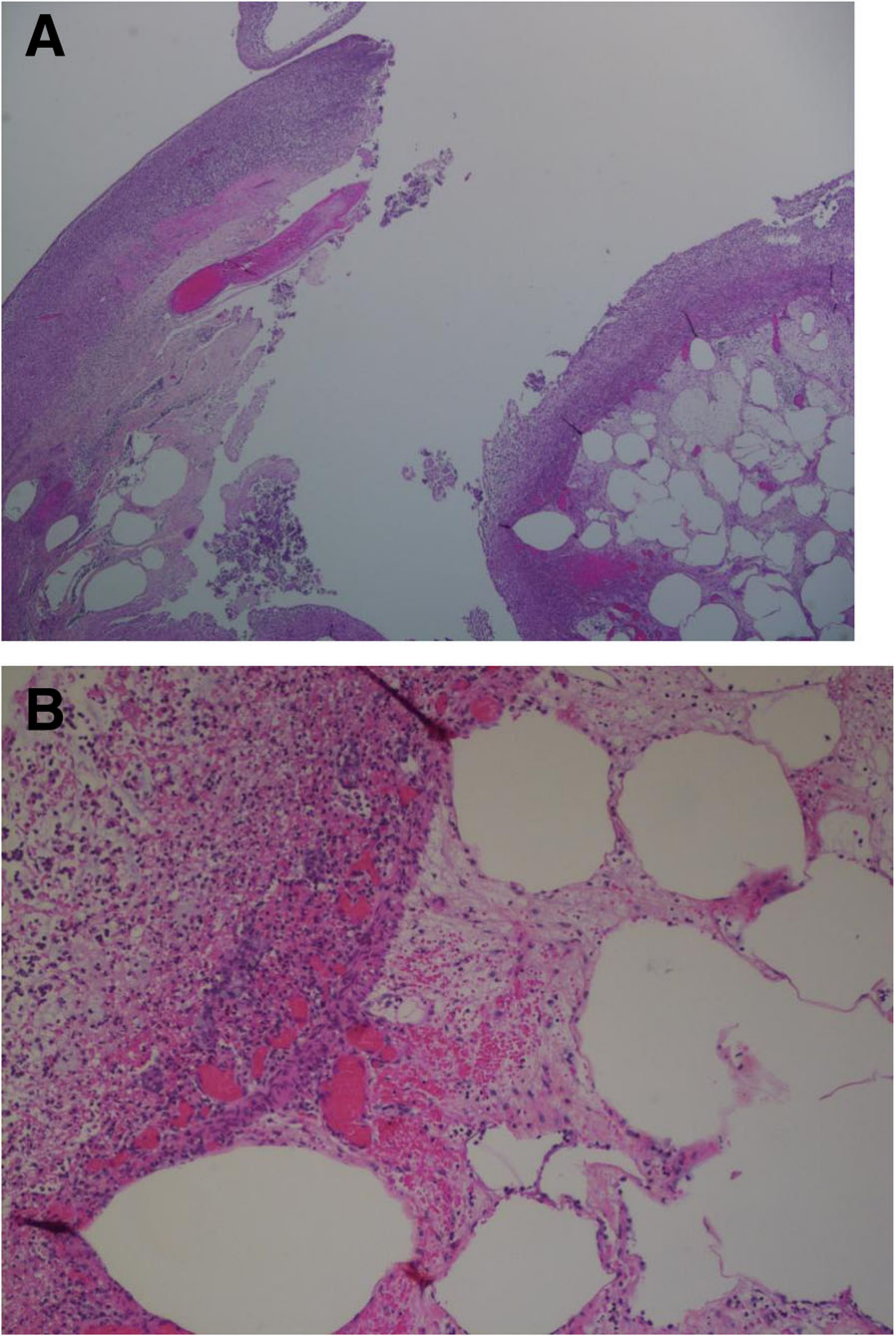
Immunohistochemical assessment of the surgical specimen. **a**. Photomicrograph of colon with necrotizing colitis and perforation site. Hematoxylin-phloxine-safranin, × 20. **b**. Photomicrograph of necrotizing colitis with submucosal pneumatosis intestinalis. Hematoxylin-phloxine-safranin, × 100

The pathological evaluation of the ileocolic resection specimen was consistent with classical features of necrotizing enterocolitis with perforation and acute peritonitis (Fig. 3). The perforation site in the colon showed transmural necrosis with heavy acute inflammation as well as numerous empty, dilated spaces in the submucosa, consistent with pneumatosis intestinalis. The rest of the resected bowel showed patchy partial (in ileum and colon) to full thickness necrosis (in appendix), transmural acute inflammation and pneumatosis intestinalis in the terminal ileum and distal colon.

The infant had an uneventful postoperative recovery. Initially her nasogastric tube was placed to low intermittent suction then transitioned to straight drainage. When the nasogastric output was minimal, she was worked up of oral feeds of expressed breast milk. She progressively reached full feeds with breastmilk and was discharged home within 2 weeks. A contrast study was performed at 7 weeks, which revealed patency of the distal colon with no evidence of stricture. Five months after her first operation, the patient underwent uncomplicated elective laparotomy for reversal of both stomas and primary anastomosis. Mild adhesions were found at repeat laparotomy with no distal atresia. She was advanced to a full p.o. feeds on postoperative day 5. The resected end-ileostomy specimen submitted for histologic evaluation, showed an unremarkable bowel tissue with no evidence of fibrosis or scarring from the neonatal episode of NEC.

## Discussion and literature review

This complex case originating from a seemingly benign initial presentation, touches on numerous points for discussion principally involving NEC in the term infant. This discussion includes a description of the pathophysiology, risk factors, presentation and complications including intestinal perforation in the neonate.

NEC is an inflammatory necrosis of the intestine that can be fatal [1, 2]. It is commonly associated with prematurity and low birth weight [1, 3]. However, since 1973 NEC has become a increasingly recognized entity in term neonates, constituting 10–20% of NEC cases [1, 4, 5]. Since that time differences between preterm and term NEC have emerged, leading to the notion that term NEC could be considered a separate clinical entity. The pathophysiology of NEC is multifactorial with a complex and delicate interplay between genetics, gut microbiome, peripartum events and nutrition encompassing only a few of the factors [3]. In preterm infants NEC involves the interaction between three main components: imbalance of intestinal microflora, damage to the intestinal wall and finally activation of the inflammatory cascade [6]. In contrast, NEC in the term infant involves a compromised intestinal blood flow, leading to ischemia and subsequent activation of the inflammatory cascade [7, 8]. Depending on the composite of risk factors present, intestinal hypoperfusion can be the result of either hemodynamic redistribution to ensure brain and heart perfusion or the result of poor tissue perfusion secondary to diastolic backflow [8].

Risk factors for term NEC can be divided into antenatal and post-natal categories [6–11]. Some of the antenatal risk factors include preeclampsia and maternal diabetes. The post-natal category can be further divided into medical aetiologies, including hypoglycemia, milk protein allergy to list a few and an organic subsection, including congenital heart disease, gastroschisis, vascular abnormalities and myelomeningocele [6–11]. Of these risk factors the most prominent, compromising over 50% of term NEC cases, is the presence of cardiac anomalies requiring surgical intervention [1]. While there are numerous factors specific to term infants, there are additional factors common to all infants regardless of gestational age at birth including: formula feeding, hypoxia, hypotension requiring inotrope support, birth asphyxia, intrauterine growth restriction, polycythemia, chorioamnionitis, exchange transfusion, umbilical lines, maternal cocaine use and severe anemia [6]. Many of these risk factors not only relate to gut perfusion but can also be linked to the final common pathway of inflammatory cascade activation that is present in both preterm and term NEC pathophysiology. The only proven protective factor against NEC is the use of human breast milk as it is postulated to help maintain the appropriate balance of gut flora [12].

Clinical presentation of term and preterm NEC are similar with a wide range in presenting symptoms from subtle and non-specific to life threatening [6]. There are two case reports of term NEC presenting with bloody stool, one case additionally presented with mild abdominal distension and the other with an increased respiratory rate with no other abnormalities noted on exam, making the diagnosis of NEC in this population challenging [13, 14]. Onset of NEC symptoms differs between the term and preterm cohorts. Preterm infants present on average from day 13–20, while term infants present earlier on average from day 5–9 [1, 6]. The caveat subgroup are term infants with a cardiac lesion awaiting surgery, as they mimic the natural history and presentation of the preterm cohort [1]. An additional difference between preterm and term NEC cases includes location of the disease, severity and overall outcome. The small intestine is more commonly affected in preterm infants while the colon is the more frequent location in term infants [8, 11, 15]. The trend is the greater the gestational age the more distal the site of intestinal involvement [15]. Broadly, the preterm infants with NEC have more severe disease when compared with term neonates [1]. Term neonates were less likely to require surgical intervention and subsequently less likely to develop NEC totalis or strictures. The survival rate for term infants with NEC Bell stage I (suspected diagnosis) is 85 and 75% for Bell stage II (definitive diagnosis) or greater [1, 6]. Overall, the severity and outcomes of term NEC are more favourable when compared to their preterm counterparts.

While perforation is less prevalent in term versus preterm NEC, it is a known complication that has a significant impact on survival. The overall incidence of gastrointestinal perforation in the NICU is 0.6%. Perforations are more common in the small intestine with transverse colon perforations being exceedingly rare [16, 17]. In the preterm population, the most frequent cause of gastrointestinal perforation is NEC [18]. The most common causes of colonic perforation include NEC, meconium ileus and Hirschsprung’s disease [17]. The overall prognosis depends on the underlying pathology, any concurrent medical issues, gestational age and birth weight [18]. Gastrointestinal perforations have a high mortality rate ranging from 40 to 70% [18]. This is a life-threatening condition that requires immediate recognition and intervention.

In reviewing this case under the lens of term NEC with intestinal perforation there are numerous novel aspects where this case contravenes previously reported cases in the literature including clinical presentation, term NEC risk factors and the location of the lesion.

In this case the infant presented with bloody stools on the second day of life. In the reported literature the average onset of symptoms for term NEC is day 5–9 days, making this a very early presentation. A more common diagnosis is Cow’s milk protein allergy (CMPA) as bloody stools are the hallmark clinical feature in non-IgE mediated CMPA reactions [19]. The average CMPA symptom onset in term infants is at 3.5 days and 23 days in premature infants [19]. Additionally, case reports of preterm infants have reported extremes in the onset of early symptoms well before the average onset, thus it is likely possible in the term neonate as well [20]. However, as this case unfolded it became markedly less likely that CMPA was responsible for the large colonic perforation as this is not a known complication of CMPA. In this case the infant did receive commercial formula three times, however given the extent of the perforation and the timeline, we postulate that the event had to have occurred earlier and was subsequently exacerbated by the introduction of formula feeds. It is plausible that the perforation was present leading to an ileus and the introduction of any milk - human or cow’s milk- may have exacerbated the local inflammatory reaction of the intestinal mucosa, intensifying the damage.

When examining this case for any potential NEC risk factors, several known risk factors can be excluded including cardiac lesions, gastroschisis and sepsis. During the surgical repair the bowel vasculature was inspected with no evident abnormalities of the watershed areas ruling out vascular malformation as a cause. The only other potential risk factor was the development of maternal hypertension and preeclampsia in the last month of pregnancy. Unfortunately, the placenta was discarded by the referring hospital and unavailable for further testing. Duci et al. looked at maternal and placental risk factors for NEC. From their analysis preeclampsia was not an independent risk factor, but indirectly involved in effecting growth restriction which places the neonate at higher risk for NEC [21]. In this case the infant was not growth restricted, so it is unlikely that the maternal preeclampsia was the inciting event.

Lastly the location of the intestinal perforation was the transverse colon. Typically, a term infant would see involvement of the distal colon to the rectum, again, making this case unique as involved a unique distribution of the proximal and transverse colon.

This case demonstrates a unique early presentation of NEC in a term neonate with an initial isolated episode of rectal bleeding in an otherwise healthy child. While initially thought to be secondary to a benign process, rapid and prudent workup revealed the true aetiology of colonic perforation secondary to NEC on day of life two. NEC in the term neonate accounts for 10–20% of all NEC and while not as common, it merits consideration when a term neonate presents with bloody stools. Term NEC presents at an average of 5–9 days of life. Atypical early presentations warrant transfer to a tertiary centre facility for a thorough workup so that the rare but ominous possibilities such as advanced NEC and intestinal perforation can be ruled out. Fortunately, in this case the potentially life-threatening aetiology was determined in a timely manner without further complication to the neonate.

## Data Availability

Not applicable.

## Abbreviations

NEC: Necrotizing enterocolitis
NICU: Neonatal intensive care unit
CMPA: Cow’s milk protein allergy
